# Exploring Longitudinal Gut Microbiome towards Metabolic Functional Changes Associated in Atopic Dermatitis in Early Childhood

**DOI:** 10.3390/biology12091262

**Published:** 2023-09-20

**Authors:** Preecha Patumcharoenpol, Amornthep Kingkaw, Massalin Nakphaichit, Pantipa Chatchatee, Narissara Suratannon, Gianni Panagiotou, Wanwipa Vongsangnak

**Affiliations:** 1 Interdisciplinary Graduate Program in Bioscience, Faculty of Science, Kasetsart University, Bangkok 10900, Thailand; preecha.pa@ku.th (P.P.); amornthep.ki@ku.th (A.K.); 2 Department of Biotechnology, Faculty of Agro-Industry, Kasetsart University, Bangkok 10900, Thailand; fagimln@ku.ac.th; 3 Center of Excellence for Allergy and Clinical Immunology, Division of Allergy, Immunology and Rheumatology, Department of Pediatrics, Faculty of Medicine, Chulalongkorn University, Bangkok 10330, Thailand; pantipa1111@yahoo.com; 4 King Chulalongkorn Memorial Hospital, The Thai Red Cross Society, Bangkok 10330, Thailand; 5 Microbiome Dynamics, Leibniz Institute for Natural Product Research and Infection Biology–Hans Knöll Institute, 07745 Jena, Germany; gianni.panagiotou@leibniz-hki.de; 6 Faculty of Biological Sciences, Friedrich Schiller University, 07743 Jena, Germany; 7 Department of Medicine, The University of Hong Kong, Hong Kong SAR, China; 8 Department of Zoology, Faculty of Science, Kasetsart University, Bangkok 10900, Thailand; 9 Omics Center for Agriculture, Bioresources, Food, and Health, Kasetsart University (OmiKU), Bangkok 10900, Thailand

**Keywords:** atopic dermatitis, early childhood, longitudinal gut microbiome, metabolic functions

## Abstract

**Simple Summary:**

Atopic dermatitis (AD) is a skin disease associated with changes in the gut microbiome early in life. We conducted a comprehensive study to investigate the gut microbiome of Thai children with AD compared to their healthy counterparts. Our study involved both longitudinal analysis, starting from 9 months of age until 30 months, and cross-sectional analysis, comparing patients in the same age group to explore temporal variation. Accordingly, differences were found in bacteria that are potentially identified to produce short-chain fatty acids, which are important for gut health. These children with AD also showed differences in certain metabolic activities related to vitamin production and host immune response. This study is the first challenge to track these gut bacteria and metabolic changes over time in Thai children with allergies. Understanding these differences can help us develop better treatments for AD and similar conditions, benefiting children’s health worldwide.

**Abstract:**

Atopic dermatitis (AD) is a prevalent inflammatory skin disease that has been associated with changes in gut microbial composition in early life. However, there are limited longitudinal studies examining the gut microbiome in AD. This study aimed to explore taxonomy and metabolic functions across longitudinal gut microbiomes associated with AD in early childhood from 9 to 30 months of age using integrative data analysis within the Thai population. Our analysis revealed that gut microbiome diversity was not different between healthy and AD groups; however, significant taxonomic differences were observed. Key gut bacteria with short-chain fatty acids (SCFAs) production potentials, such as *Anaerostipes*, *Butyricicoccus*, *Ruminococcus*, and *Lactobacillus* species, showed a higher abundance in the AD group. In addition, metabolic alterations between the healthy and AD groups associated with vitamin production and host immune response, such as biosynthesis of menaquinol, succinate, and (Kdo)2-lipid A, were observed. This study serves as the first framework for monitoring longitudinal microbial imbalances and metabolic functions associated with allergic diseases in Thai children during early childhood.

## 1. Introduction

Atopic dermatitis (AD) is the most common chronic inflammatory skin disorder that affects up to 20% of children worldwide [1,2]. The prevalence of AD in children is also on the rise worldwide, which develops from a complex interplay between environmental, genetic, immunologic, and biochemical factors [3,4,5]. AD is a multifaceted disease affecting patients with epidermal barrier dysfunction and dry and sensitive skin. AD frequently presents with monotonous eczematous lesions on the face, neck, and skin folds, and it may also present with other features [6,7,8]. The clinical trials involving targeted therapies, such as Dupilumab [9,10] and Upadacitinib [11,12,13], have previously demonstrated an alleviation of AD symptoms. Over the last decade, numerous studies reported that gut microbiome perturbation during infancy potentially contributes to allergic diseases [2,14,15,16,17]. Recent investigations have observed longitudinal changes in gut microbiome in children with AD compared to healthy groups [18,19,20,21]. Common findings among those studies include gut microbiome alterations related to host immune development and short-chain fatty acids (SCFAs) production-related bacteria, such as *Bifidobacterium*, *Bacteroides*, *Ruminococcaceae*, and *Lachnospiraceae*. In addition, many studies reported an association between SCFAs level and AD [18,22,23,24]. Ta et al. (2020) identified that allergen-sensitized AD is associated with a decreased level of SCFAs, e.g., acetate, butyrate, and propionate, along with a depletion of gene expression related to glycolysis, butyrate, and propionate biosynthesis pathways [22]. Cait et al. (2019) reported a significant depletion of CAZymes and butyrate-producing genes of the children who had atopic disease [23].

A comprehensive understanding of the dynamics and features of the human gut microbiome across the longitudinal gut microbiome towards metabolic functional changes has become an essential area of research for alternative therapeutic avenues of relevant co-morbidities. So far, 16S rRNA gene sequencing and shotgun metagenomics have become increasingly feasible, allowing not only for the retrieval of taxonomic information, but also metabolic functions of the gut microbiome. Moreover, bioinformatics tools, such as the MetGEMs toolbox [25] and PICRUSt2 [26], allow for the prediction of the metabolic functional abundance of a microbial community based on 16S rRNA gene sequencing profiles. Furthermore, metaproteomics has also emerged as a powerful tool for identifying and quantifying all expressed proteins from microbial communities, which gives insight into the activities of microbial communities at the molecular level [27]. 

Our study aimed to explore taxonomy and metabolic functions across longitudinal gut microbiomes associated with AD in early childhood using integrative data analysis within the Thai population. Initially, 16S rRNA gene sequencing was used to study longitudinal gut microbiome of the early childhood population (<3 years old) from a Thai population-based allergy birth cohort study. Integrative analysis of metagenomic and metaproteomic data was then performed for identification of key bacteria, metabolic functions, and pathways related to AD pathogenesis. This pilot study marks one of the early efforts for monitoring the longitudinal gut microbial community and their metabolic functions in relation to AD. This study contributes to a better understanding of AD pathogenesis and offers insights for potential therapeutic strategies, as well as an avenue for preventing allergic disease development within the Thai population.

## 2. Materials and Methods

### 2.1. Study Design and Fecal Sample Collection 

This study used the fecal samples from the population-based birth cohort study, conducted at King Chulalongkorn Memorial Hospital, the Thai Red Cross Society, Bangkok, Thailand. The study was approved by the Ethics Committee of the Faculty of Medicine, Chulalongkorn University, Bangkok, Thailand, under the approval reference number 358/58. Parents of subjects agreed to participate in the study. Written informed consent was obtained from the parents or guardians of the participants before collecting clinical data and fecal samples. This research was performed according to the Helsinki Guidelines.

To be eligible for inclusion in this study, healthy full-term infants born to healthy pregnant women were selected. Exclusion criteria encompassed the factors that may influence gut microbiome, including administration of antibiotics to the infants within 1 month prior to the collection of the fecal sample; presence of other medical conditions except allergies in the mothers, such as hypertension, diabetes, liver disease, thyroid disease, or mental problems deemed inappropriate by physicians. For the infants, the exclusion criteria included those with serious medical conditions and congenital anomalies. 

For fecal sample collection, fecal samples from 62 enrolled participants from 9 to 30 months of age were collected, of which 39 participants were healthy (control) and 23 participants were diagnosed with AD. In total, 139 fecal samples were collected, which included 9–12 months (*n* = 31 control, *n* = 19 AD), 18–21 months (*n* = 36 control, *n* = 17 AD), and 24–30 months (*n* = 25 control, *n* = 11 AD) (Supplementary Table S1).

### 2.2. Clinical Data Collection 

Clinical data during the perinatal and postnatal periods were collected through interviews by physicians and study nurses. These include family history of atopic diseases (atopic dermatitis, allergic rhinitis, or asthma in a parent or siblings), family income, delivery mode, sex, duration of breastfeeding, exposure to pets, history of illnesses, and antibiotic use. To diagnose AD, allergy specialists performed a detailed history taking and physical examination, following the criteria set by the American Academy of Dermatology [28].

### 2.3. Fecal Sample Preparation 

Fecal samples were collected from enrolled participants in this study. A 20 g fecal sample was collected from the diaper and placed into a sterile container (30 × 117 mm) and immediately placed on ice for transferring to storage at −80 °C. Fecal samples were prepared following the protocol established by Kisuse et al. [29]. The fecal samples were diluted ten-fold with phosphate-buffered saline (pH 8.0) using a stomacher blender (Stomacher^®^ 80 Biomaster, Seward, Worthing, UK) for 5 min. In following, 1 mL of fecal slurry was placed into a 1.5 mL centrifuge tube and then stored at −80 °C until further analysis.

### 2.4. Microbial DNA Extraction and 16S rRNA Gene Sequencing 

The microbial DNA was extracted using a combined bead meter method and a QIAamp^®^ DNA stool mini kit (Qiagen GmbH, Hilden, Germany), following the protocol described by Kisuse et al. [29]. After the extraction, the DNA was quantified and evaluated for quality using a Nanodrop spectrophotometer (Thermo Fisher Scientific, Waltham, MA, USA) and was immediately stored at −20 °C. The fecal microbiome was analyzed using 16S rRNA gene sequencing based on the method described by Sathikowitchai et al. Briefly, the V3-V4 variable region of the 16S rRNA gene was amplified using the forward primer Imina V3-V4-F (5′-TCGTCGGCAGCGTCAGATGTGTATAAGAGACAGCCTACGGGNGGCWGCAG-3′) and the reverse primer Imina-V3-V4-R (5′-GTCTCGTGGGCTCGGAGATGTGTATAAGAGACAGGACTACTACHVGGGTATCTAATCC-3′). The cycling conditions consisted of an initial denaturation at 94 °C for 2 min, followed by 25 cycles of denaturation at 94 °C for 20 s, annealing at 57 °C for 30 s, and extension at 72 °C for 30 s, and a final extension at 72 °C for 10 min. The PCR products were purified using NucleoSpin^®^ Gel and PCR Clean-up (MACHEREY-NAGEL Inc., Allentown, PA, USA) according to the manufacturer’s protocol. The 16S rRNA amplicon sequencing was performed using the Illumina MiSeq platform (Illumina, San Diego, CA, USA).

### 2.5. Microbiome Data Processing

The 16S amplicon paired-end sequence data were processed by fastp [30] to remove low-quality reads and reads with ambiguous nucleotides. The paired-end sequence data were trimmed for the last 10 nucleotides, and the primers at the 5′ of reads were also trimmed as a quality control measure. The remaining high-quality pair reads were then denoised and merged into amplicon sequence variants (ASVs) using the DADA2 pipeline (v.1.10) [31] with default parameters. ASV taxonomic assignment was performed using QIIME2’s naïve bayes classifier (v.2021.8) [32] with the SILVA 99% OTU database v.138 [33] using 70% cut-off. Microbial taxa abundance was preprocessed and filtered by the removal of ASVs with no phylum classification or relative low prevalence (<10 samples). In addition, a removal of singleton ASVs was performed to reduce a potential artifact from sequencing errors.

### 2.6. Microbial Taxonomy, Functional Composition, and Integrative Meta-Omics Analysis

Microbiome data were analyzed for microbial diversity, taxonomic, and functional analysis for both healthy (control) and AD groups across three time points i.e., 9–12 months, 18–21 months, and 24–30 months. The alpha diversity was calculated using Chao index, Shannon’s index, and Simpson’s index. The beta diversity was calculated as a Bray–Curtis, UniFrac, and Jaccard distance. Associations between diversity values and sample conditions (control or AD) were calculated using linear regression for alpha diversity and ADONIS for beta diversity.

Significant differences in microbial taxonomy abundance between control and AD groups were identified using ANCOM-BC (v.1.40) [34]. For longitudinal analysis, confounding factors, such as time point, family income, sequencing batch, or exposure to pet were included as covariates. For cross-sectional analysis, fecal samples at each time point were compared between control and AD groups using ANCOM-BC upon covariates of family income, sequencing batch, or exposure to pet. 

Concerning functional compositions, PICRUSt2 [26] and MetGEMs [25] were initially utilized to predict KO IDs and metabolic pathways abundance in each sample. Subsequently, the significant abundance differences of KO IDs and metabolic pathways between the control and AD groups were identified using ANCOM-BC. Notably, the same covariates as earlier descriptions were used for longitudinal and cross-sectional analysis, respectively.

To further identify the potential metabolic routes, significant metabolic pathway results from PICRUSt2 were selected and then mapped with metaproteomic datasets from Kingkaw et al. (2020) [27] by EC number. The top functional contribution was ranked and visualized by predominant microbial groups with related KO IDs and EC numbers.

## 3. Results and Discussion

### 3.1. Assessment of Participant Characteristics

As noticed in Table 1, 39 participants were healthy (control) and 23 participants were diagnosed with AD. Within the AD group, 19 individuals had mild AD (SCORAD index < 25), while 4 individuals had moderate AD (SCORAD index between 25 and 50). Across the AD group, 15 individuals experienced remission before reaching 30 months, while 8 individuals had persistent AD beyond 30 months of age. It is noted that there are no other co-morbidities, such as food allergy or asthma, which were observed in the AD group. Furthermore, there were no significant differences in demographic characteristics between the two groups (Table 1) and no significant changes over time, as determined by Fisher’s exact test (Supplementary Table S1). However, we observed that participants in the control group had greater exposure to pets (i.e., dogs and cats) than those in the AD group (*p*-value < 0.05), which aligns with previous reports linking pet exposure to a lower risk of development of atopic disease [35,36,37]. 

### 3.2. Differential Shifts in Gut Microbial Diversity Trajectories over Time between Healthy and AD Participants

**Figure 1:**
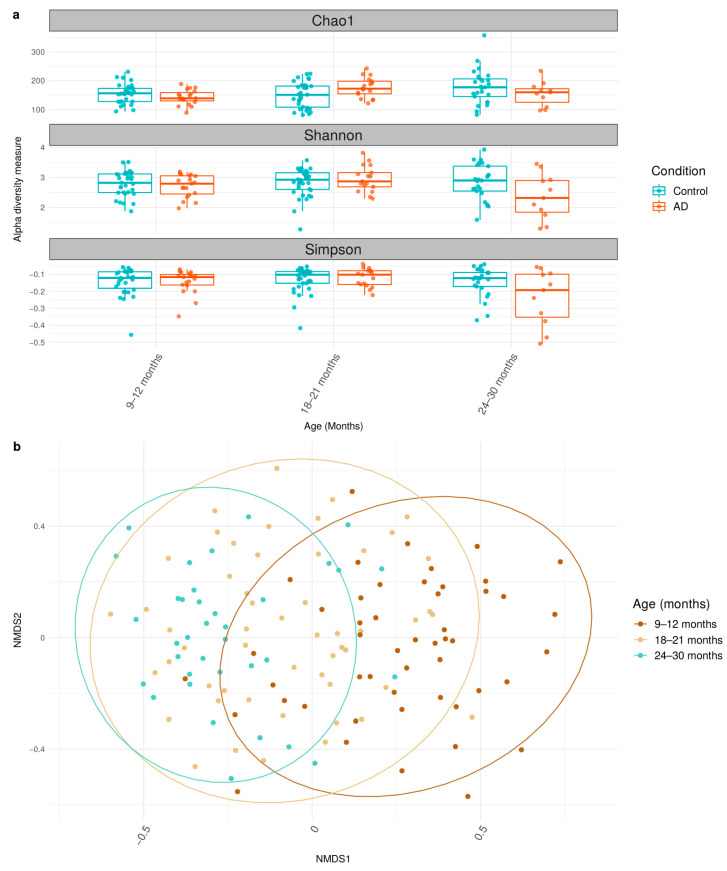
Alpha and beta diversity of gut microbiome between control and AD groups across 9–30 months. (**a**) Boxplot shows the median and interquartile range (IQR) of diversity values between control and AD groups. (**b**) Bray NMDS plots across 9–12 months, 18–21 months, and 24–30 months are illustrated. The statistically significant data under *p*-value < 0.05 are considered and available in Supplementary Tables S4 and S5.

To determine gut microbial diversity of each sample, alpha diversity indices (Chao1, Shannon, and Simpson) were calculated using 16S rRNA genes data and compared between control and AD groups using linear modelling (Figure 1a, Supplementary Table S2). Chao1, Shannon, and Simpson indices did not display any statistically significant shifts in microbial diversity across all time points (9–30 months) in both control and AD groups (Supplementary Table S2). Previous studies, such as Lee et al. (2022) and Ismail et al. (2012) [18,38], reported a decrease of bacterial diversity among AD infants in their respective studies. Notably, our findings did not show those observations (Figure 1a). 

Furthermore, the beta diversity using Bray distance was analyzed using ADONIS (Figure 1b, Supplementary Table S3). The analysis showed that the patterns of microbiome gradually shifted over time among participants (age, R^2^ = 0.043; *p*-value < 0.05). The result from ADONIS also suggested that relevant factors, such as income and exposure to pets, impact the microbiome’s structure. Overall, these results support the notion of time-dependent gut microbial diversity of healthy and AD Thai children during early childhood. 

### 3.3. Comparison of Bacteria Abundances in Healthy and AD Participants 

**Figure 2:**
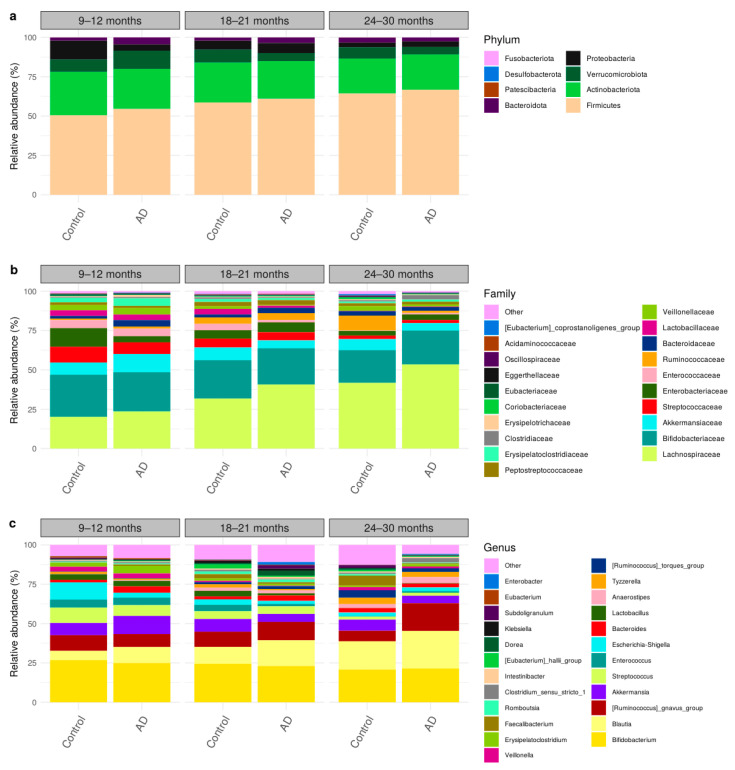
Taxonomic compositions of gut microbiome using longitudinal sampling in early childhood (9–30 months) at (**a**) phylum, (**b**) family, and (**c**) genus levels.

Taxonomic compositions of all participants in the control and AD groups were classified into 8 phyla, 70 families, and 199 genera, as shown in Figure 2. The three major prevalent phyla, based on their relative abundance, were Firmicutes (56%), Actinobacteriota (28%), and Proteobacteria (5%). Interestingly, these phyla were dominated by single families, namely *Lachnospiraceae* (32%), *Bifidobacteriaceae* (27%), and *Enterobacteriaceae* (5%), respectively. These taxonomic abundances are similar to the gut microbiome composition among Asian children [29,39]. As shown in the relative abundance over the time (Figure 2), Firmicutes increased, while Actinobacteriota and Proteobacteria decreased in both control and AD groups, which is in agreement with typical gut microbiome progression in healthy children [39,40].

Considering gut microbiome changes between the control and AD groups, ANCOM-BC analysis revealed that two bacterial families and three bacterial genera were significantly associated with AD (Table 2 and Supplementary Table S4). At the family level, *Lachnospiraceae* and *Butyricicoccaceae* were significantly enriched in the AD group. At the genus level, *Ruminococcus*, *Anaerostipes,* and *Butyricicoccus* were also found to be significantly in higher abundance with the AD group. Interestingly, prior studies have associated the bacteria, i.e., *Anaerostripes hadrus* and *Ruminococcus gnavus,* with inflammatory diseases [41,42,43,44] and being enriched in AD [18,45]. Similarly, *Butyricicoccus* has also been found to be enriched in AD condition [46]. Our result supports their associations by further identifying specific species of bacteria or functional genes.

**Figure 3:**
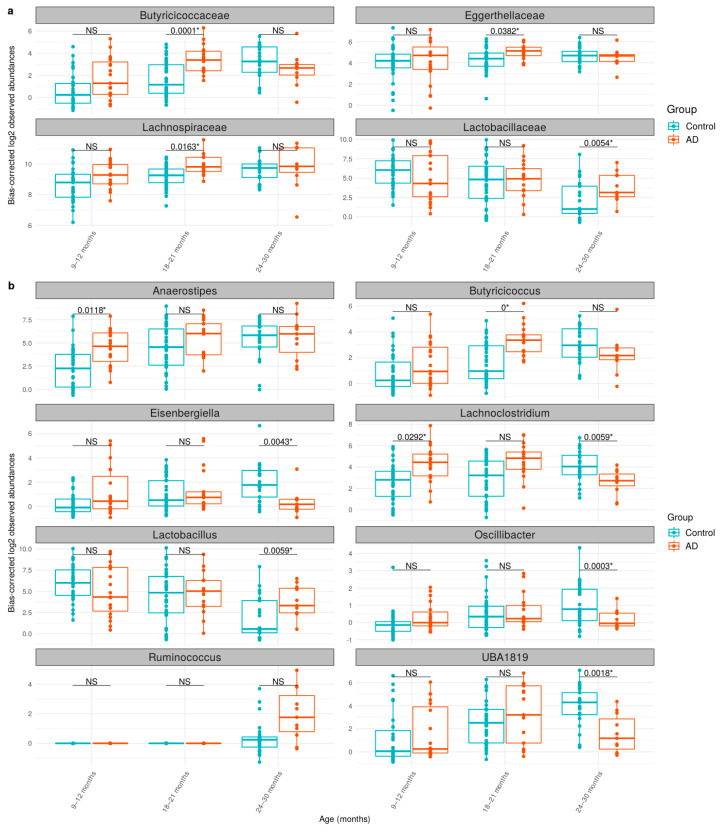
The taxonomic abundances of the gut microbiome between control and AD groups at (**a**) family and (**b**) genus levels. The log2 observed abundance was corrected by ANCOM-BC. The boxplot shows the median abundances and interquartile range (IQR) of bacterial taxa. NS indicates no significance (q-value > 0.05), whereas * indicates a cross-sectional significant difference in abundance between the control and AD groups. The cross-sectional analysis was performed by ANCOM-BC (see Section 2.6).

Cross-sectional analysis between control and AD groups is shown in Figure 3, which displays the significantly different bacterial communities at the family and genus levels between the control and AD groups at different time points (Figure 3a,b). At ages of 9–12 months, *Anaerostipes* and *Lachnoclostridium* were over-represented in the AD group. At ages of 18–21 months, *Butyricicoccaceae*, *Eggerthellaceae*, *Lachnospiraceae*, and *Butyricicoccus* were more abundant in the AD group. At the age of 24–30 months, *Lactobacillus* was shown to be higher in the AD group, while *Eisenbergiella*, *Oscillibacter*, *Lachnoclostridium*, or *UBA1819* were found to be enriched in the control group. As shown in Table 3 and Supplementary Table S5, the increasing abundance of *Oscillibacter* was notably found at 24–30 months, indicating a cross-sectional variation occurrence as described in other studies, of which *Oscillibacter* was enriched in non-AD in early childhood [18,47], although its purpose was unclear. In our study, *Lachnoclostridium* also exhibited an interesting pattern upon cross-sectional variation (Figure 3 and Table 3), where it was significantly enriched in the control group at 24–30 months. *Lachnoclostridium* were characterized with lipid metabolism and influence on SCFAs level [48], but its effects on AD pathogenesis have yet been described.

### 3.4. Metabolic Functional Compositions of Longitudinal Gut Microbiome

The microbial functional compositions were predicted by PICRUSt2, followed by longitudinal analysis using ANCOM-BC. As listed in Table 4, the identified eight significant metabolic pathways that were enriched in the AD group are the superpathway of menaquinol-9 biosynthesis (PWY-5845), superpathway of menaquinol-6 biosynthesis I (PWY-5850), superpathway of menaquinol-10 biosynthesis (PWY-5896), superpathway of demethylmenaquinol-6 biosynthesis I (PWY-5860), superpathway of demethylmenaquinol-9 biosynthesis (PWY-5862), superpathway of (Kdo)_2_-lipid A biosynthesis (KDO-NAGLIPASYN-PWY), superpathway of histidine, purine, and pyrimidine biosynthesis (PRPP-PWY), and TCA cycle IV (2-oxoglutarate decarboxylase) (P105-PWY). Additionally, MetGEMs analysis showed several pathways were enriched in the control group, mainly glycolysis III (from glucose) (ANAGLYCOLYSIS-PWY), 5-aminoimidazole ribonucleotide biosynthesis I (PWY-6121), L-arginine biosynthesis II (acetyl cycle) (ARGSYNBSUB-PWY), and superpathway of L-aspartate and L-asparagine biosynthesis (ASPASN-PWY). For other metabolic pathways prediction, MetGEMs prediction, followed by longitudinal analysis with ANCOM-BC, was performed. However, significant difference in pathways or KO IDs between the groups was not observed. Supplementary Tables S6–S11 show full results of longitudinal and cross-sectional analysis of metabolic functional compositions in gut microbiome.

As shown in Table 4, we identified that many pathways related to biosynthesis of menaquinol and demethylmenaquinol were significantly higher in abundance in the AD groups. This finding is consistent with our earlier study [27], where demethylmenaquinone methyltransferase (DMM, EC: 2.1.1.163) was uniquely expressed in samples from the AD group where DMM was involved in the conversion of demethylmenaquinol (e.g., demethylmenaquinol-6 or demethylmenaquinol-9) to menaquinol (e.g., menaquinol-6 or menaquinol-9), respectively. Taken together, the results suggest that gut bacteria could produce menaquinol in the long run, which may be an alternative source of vitamins in patients.

Moreover, the result indicated that the biosynthesis of (Kdo)_2_-lipid A was enriched in the AD group. (Kdo)_2_-lipid A (3-deoxy-d-manno-octulosonic acid-lipid A) is the essential component of lipopolysaccharide in most Gram-negative bacteria, such as *E. coli* K12 and related Proteobacteria, which are pathogenic bacteria [49] that serve as a strategy to modulate bacterial virulence as well as to avoid recognition by the mammalian innate immune systems. Furthermore, the biosynthesis of histidine, purine, and pyrimidine and TCA cycle IV (2-oxoglutarate dehydrogenase) were also majorly enriched in the AD group. Regarding histidine, purine, and pyrimidine biosynthesis, it requires phosphoribosylpyrophosphate (PRPP), thus PRPP is regarded as a precursor for the synthesis of nucleic acids, proteins, and for the NAD(P) coenzymes. Upon integrative metaproteomic data of higher protein expression of PRPP synthetase being observed in AD [27], this suggests that the formation of PRPP might be associated with metabolic control of AD patients. For TCA cycle IV, the absence of 2-oxoglutarate dehydrogenase complex (EC: 1.2.1.105) was found, which could notionally result in incomplete oxidative or reductive TCA cycles that supply biosynthetic intermediates, e.g., succinate in response to AD condition. This enzyme complex is essential for succinate formation, which is a key precursor and plays an important role in either propionate or acetyl-CoA formation, which can then be converted to acetate or butyrate formation [50,51,52,53].

In addition to metabolic pathways related to longitudinal gut microbiome, other metabolic pathways have also been identified to vary temporally. As shown in Table 5, the ANCOM-BC cross-sectional analysis on PICRUSt2’s prediction identified the superpathway of histidine, purine, and pyrimidine biosynthesis (PRPP-PWY) and allantoin degradation IV (anaerobic) (PWY0-41) to be more abundant in the AD groups (18–21 months and 24–30 months). Notably, the enrichment of histidine, purine, and pyrimidine biosynthesis (PRPP-PWY) in the AD group supports that the formation of PRPP might be needed for metabolic control of AD patients. Additionally, the AD group exhibited an enriched presence of alpha-galactosidase (K07406) and allantoin degradation IV (anaerobic) (PWY0-41). 

Furthermore, ANCOM-BC cross-sectional analysis on MetGEMs’s prediction revealed significant differences between control and AD groups in the other 3 pathways and 12 KO IDs. At 18–21 months, pathways of purine ribonucleosides degradation (PWY0-1296) and superpathways of pyrimidine deoxyribonucleoside salvage (PWY-7200) and adenine and adenosine salvage III (PWY-6609) were significantly enriched in control, AD, and control groups, respectively. Undecaprenyl diphosphate synthase (K00806) was also found to be enriched in the AD group at this time point. At 24–30 months, the AD group had enriched in hydroxymethylpyrimidine/phosphomethylpyrimidine kinases (K14153, K00877, K00941), succinate dehydrogenase subunits (K00239, K00240, K00241, K00242), and fumarate reductase subunits (K00244, K00245, K00246, and K00247).

### 3.5. Identification of Potential Metabolic Routes and Associated Bacteria Genera in AD Using Integrated Metagenomic and Metaproteomic Approaches

**Figure 4:**
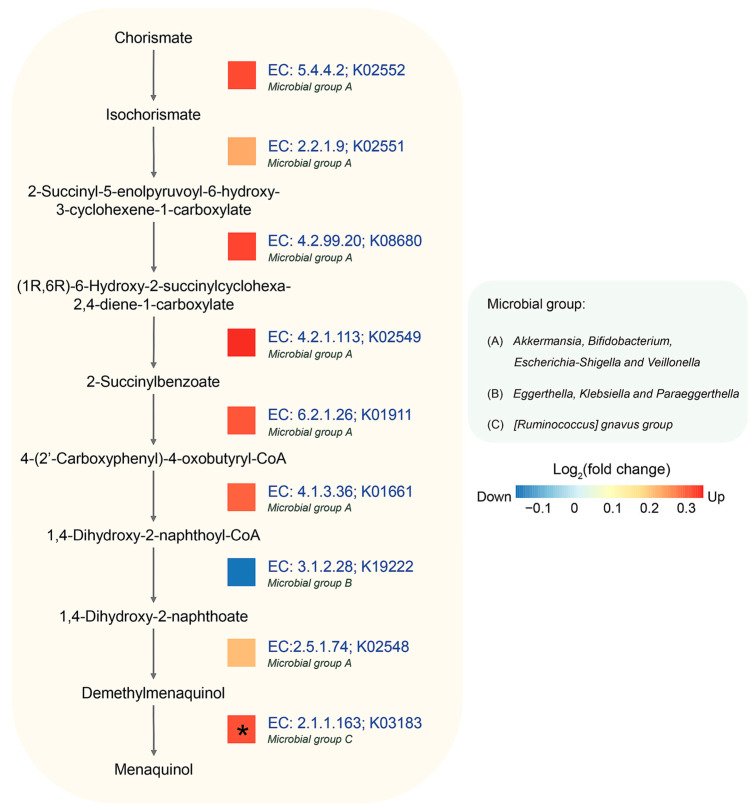
Longitudinal difference in abundance between control and AD groups in menaquinol biosynthesis using PICRUSt2. Log2 (fold change) of predicted gene abundance is visualized. The predominant microbial groups with relevant KO IDs and EC numbers were predicted by PICRUSt2. An asterisk (*) indicates a statistically significant difference in abundance based on reported metaproteomic data (Kingkaw et al., 2020 [27]).

Considering the longitudinal results, the menaquinol biosynthesis pathway showed a significant difference between control and AD groups, as shown in Figure 4. This pathway involves nine enzymes, with eight of them (EC: 5.4.4.2, EC: 2.2.1.9, EC: 4.2.99.20, EC: 4.2.1.113, EC: 6.2.1.26, EC: 4.1.3.36, EC: 2.5.1.74, and EC: 2.1.1.163) being more abundant in the AD group, though each enzyme was not significant under statistical test. From the PICRUSt2 analysis, eight genera, i.e., *Akkermansia*, *Bifidobactrium*, *Escherichia-Shigella*, *Veillonella*, *Eggerthella*, *Klebsiella*, *Paraeggerthella,* and *[Ruminococus] gnavus group,* were observed to be the biggest contributors to this pathway. Of these, four predominant genera, i.e., *Akkermansia*, *Bifidobactrium*, *Escherichia-Shigella*, and *Veilonella,* were found to contribute most enzymes. Notably, these genera are known for association with health and immune system of host [51,54,55,56,57]. Conversely, two enzymes, EC: 3.1.1.28 (1,4-dihydroxy-2-naphthoyl-CoA hydrolase) and EC. 2.1.1.163 (demethylmenaquinone methyltransferase, DMM), were mostly contributed by *Eggerthella*, *Klebsiella*, *Paraeggerthella,* and *[Ruminococus] gnavus group*.

After mapping the result of metaproteomic datasets, it was observed that the AD group also exhibited a significant increase in DMM. This enzyme is involved in the last step of menaquinone biosynthesis by catalyzing the methylation of demethylmenaquinone using S-adenosylmethionine, resulting in the formation of menaquinone [58]. It can be inferred that the relationship between menaquinone and the gut microbiome might play important roles in the mechanism of AD [59].

## 4. Conclusions

Our finding demonstrated the variations in both taxonomy and metabolic functions within the longitudinal Thai gut microbiome during early childhood. Key gut bacteria implicated in SCFAs production potentials, e.g., *Anaerostipes*, *Butyricicoccus*, *Ruminococcus*, and *Lactobacillus*, were identified. Additionally, we highlighted metabolic pathways associated with AD, including menaquinol biosynthesis, demethylmenaquinol biosynthesis, and (Kdo)_2_-lipid A biosynthesis; histidine, purine, and pyrimidine biosynthesis; and TCA cycle IV (2-oxoglutarate decarboxylase). These microbial imbalances may play a role in the pathogenesis of AD, emphasizing the need for further research on cutting-edge technologies for meta-omics and advanced bioinformatics tools and databases for the intricate relationships between the gut microbiome and AD. This study serves as the first framework for the monitoring of longitudinal gut microbial community-wide metabolic functions associated with allergic diseases in a Thai population-based allergy birth cohort.

## Figures and Tables

**Table 1:** Demographic characteristics of study participants.

	Control (*n* = 39)	AD (*n* = 23)	*p*-Value ^&^
Sex			0.60
Male	23 (59%)	15 (65%)	
Female	16 (41%)	8 (35%)	
Delivery method			1.00
Caesarean	14 (36%)	8 (35%)	
Vaginal	25 (64%)	15 (65%)	
Family income (monthly, THB *)			0.11
≤50,000	33 (85%)	15 (65%)	
≥50,000	6 (15%)	8 (35%)	
Birth weight			0.52
mean ± sd	3228 ± 412	3135 ± 621	
Exposure to pet			0.04
Yes	15 (38%)	6 (26%)	
No	24 (62%)	17 (74%)	
Mother history of AD			1.00
Yes	6 (15%)	3 (13%)	
No	33 (85%)	20 (87%)	

Note: Significant difference was considered under *p*-value < 0.01. * 50,000 THB = 1467.14 USD (as of 14 April 2023). ^&^ Fisher’s exact test was used to assess the difference between control and AD groups, except for birth weight, where the Welch two-sample *t*-test was applied.

**Table 2:** List of significantly different taxa across longitudinal gut microbiome between control and AD.

Taxonomic Level	Taxonomic Name	q-Value	Log2FC *
Order	*Lachnospirales*	0.0006	1.03
Family	*Butyricicoccaceae*	0.0036	1.56
Family	*Lachnospiraceae*	0.0133	0.87
Genus	*Anaerostipes*	0.0077	2.11
Genus	*Ruminococcus*	0.0292	0.99
Genus	*Butyricicoccus*	0.0386	1.39

Note: The adjusted *p*-value (q-value) was calculated under the Holm–Bonferroni correction method. A list of significantly different taxa was considered under q-value < 0.05. * Positive values represent a higher abundance in the AD group.

**Table 3:** List of significantly different taxa across gut microbiome between control and AD in cross-sectional samples.

Age	Taxonomic Level	Taxonomic Name	q-Value	Log2FC *
9–12 months	Genus	*Anaerostipes*	0.0118	3.18
	Genus	*Lachnoclostridium*	0.0292	2.56
18–21 months	Family	*Butyricicoccaceae*	0.0001	2.60
	Family	*Lachnospiraceae*	0.0163	1.02
	Family	*Eggerthellaceae*	0.0382	1.12
	Genus	*Butyricicoccus*	0.0000	2.66
24–30 months	Family	*Lactobacillaceae*	0.0054	3.49
	Genus	*Oscillibacter*	0.0003	−1.62
	Genus	*UBA1819*	0.0018	−3.84
	Genus	*Eisenbergiella*	0.0043	−2.26
	Genus	*Lactobacillus*	0.0059	3.70
	Genus	*Lachnoclostridium*	0.0059	−2.52

Note: The adjusted *p*-value (q-value) was calculated under Holm–Bonferroni correction method. A list of significantly different taxa was considered under q-value < 0.05. * A positive sign indicates higher abundance in the AD group while a negative sign indicates higher abundance in the control.

**Table 4:** List of significantly different metabolic pathways in longitudinal gut microbiome between control and AD.

Metabolic Pathways-Based MetaCyc	q-Value	Log2FC *
superpathway of menaquinol-9 biosynthesis (PWY-5845)	0.0033	0.49
superpathway of menaquinol-6 biosynthesis I (PWY-5850)	0.0033	0.49
superpathway of menaquinol-10 biosynthesis (PWY-5896)	0.0033	0.49
superpathway of demethylmenaquinol-6 biosynthesis I (PWY-5860)	0.0166	0.50
superpathway of demethylmenaquinol-9 biosynthesis (PWY-5862)	0.0166	0.50
superpathway of (Kdo)2-lipid A biosynthesis (KDO-NAGLIPASYN-PWY)	0.0225	0.69
superpathway of histidine, purine, and pyrimidine biosynthesis (PRPP-PWY)	0.0287	0.38
TCA cycle IV (2-oxoglutarate decarboxylase) (P105-PWY)	0.0390	0.65

Note: The adjusted *p*-value (q-value) and Log2FC were calculated using ANCOM-BC. A list of significantly different taxa was considered under q-value < 0.05. * A positive sign indicates higher abundance in the AD group while a negative sign indicates higher abundance in the control.

**Table 5:** List of significantly different metabolic pathways across gut microbiome between control and AD in cross-sectional samples.

Age	Metabolic Pathways-Based MetaCyc/ KO IDs	q-Value	Log2FC *
PICRUSt2			
18–21 months	superpathway of histidine, purine, and pyrimidine biosynthesis (PRPP-PWY)	0.0461	0.53
24–30 months	allantoin degradation IV (anaerobic) (PWY0-41)	0.0027	2.01
	alpha-galactosidase (K07406)	0.0195	1.20
	uncharacterized protein (K07033)	0.0468	1.28
MetGEMs			
18–21 months	purine ribonucleosides degradation (PWY0-1296)	0.0275	−0.58
	superpathway of pyrimidine deoxyribonucleoside salvage (PWY-7200)	0.0303	0.48
	adenine and adenosine salvage III (PWY-6609)	0.0390	−0.63
	undecaprenyl diphosphate synthase (K00806)	0.0132	0.74
24–30 months	hydroxymethylpyrimidine kinase/phosphomethylpyrimidine kinase/thiamine-phosphate diphosphorylase(K14153)	0.0023	0.32
	hydroxymethylpyrimidine/phosphomethylpyrimidine kinase/thiaminase (K00877)	0.0179	0.38
	hydroxymethylpyrimidine/phosphomethylpyrimidine kinase (K00941)	0.0179	0.38
	succinate dehydrogenase flavoprotein subunit (K00239)	0.0270	0.46
	succinate dehydrogenase iron-sulfur subunit (K00240)	0.0270	0.46
	succinate dehydrogenase cytochrome b subunit (K00241)	0.0270	0.46
	succinate dehydrogenase membrane anchor subunit (K00242)	0.0270	0.46
	fumarate reductase flavoprotein subunit (K00244)	0.0270	0.46
	fumarate reductase iron-sulfur subunit (K00245)	0.0270	0.46
	fumarate reductase subunit C (K00246)	0.0270	0.46
	fumarate reductase subunit D (K00247)	0.0270	0.46

Note: Significant metabolic pathways are under q-value < 0.05. * A positive sign indicates higher abundance in the AD group while a negative sign indicates higher abundance in the control.

## Data Availability

16S rRNA gene sequencing data used in this study are available in the NCBI Sequence Read Archive (SRA) under BioProject ID PRJNA716451. Information on age, gender, family history, pet, and mode of delivery are available upon request.

## Supplementary Materials

The following supporting information can be downloaded at: https://www.mdpi.com/article/10.3390/biology12091262/s1, Table S1: Number of samples analyzed and sorted by age group, condition, and data type; Table S2: Linear regression analysis of alpha diversity; Table S3: Beta diversity analysis with ADONIS; Table S4: Results of abundance analysis with ANCOM-BC; Table S5: Results of abundance analysis with ANCOM-BC, sorted by sample’s age; Table S6: MetaCyc pathway prediction of the microbiome by PICRUSt2 of the AD and control groups; Table S7: MetaCyc pathway prediction of the microbiome by PICRUSt2 of the AD and control groups, sorted by sample’s age; Table S8: KO IDs prediction of the microbiome by PICRUSt2, sorted by sample’s age; Table S9: MetaCyc pathway prediction of the microbiome by MetGEMs of the AD and control groups; Table S10: MetaCyc pathway prediction of the microbiome by MetGEMs, sorted by sample’s age; Table S11: KO IDs prediction of the microbiome by MetGEMs, sorted by sample’s age.
